# Relative bioavailability of iron and folic acid from a new powdered supplement compared to a traditional tablet in pregnant women

**DOI:** 10.1186/1471-2393-9-33

**Published:** 2009-07-27

**Authors:** Brenda Hartman-Craven, Anna Christofides, Deborah L O'Connor, Stanley Zlotkin

**Affiliations:** 1 Department of Nutritional Sciences, Faculty of Medicine, University of Toronto, Toronto, Canada; 2 Department of Paediatrics and the Department of Public Health Sciences, Faculty of Medicine, University of Toronto, Toronto, Canada; 3 Program in Child Health and Evaluative Sciences, Research Institute, Hospital for Sick Children, Toronto, Canada; 4 Physiology and Experimental Medicine, and the Department of Clinical Dietetics, Hospital for Sick Children, Toronto, Canada

## Abstract

**Background:**

Deficiencies of iron and folic acid during pregnancy can lead to adverse outcomes for the fetus, thus supplements are recommended. Adherence to current tablet-based supplements is documented to be poor. Recently a powdered form of micronutrients has been developed which may decrease side-effects and thus improve adherence. However, before testing the efficacy of the supplement as an alternate choice for supplementation during pregnancy, the bioavailability of the iron needs to be determined. Our objective was to measure the relative bioavailability of iron and folic acid from a powdered supplement that can be sprinkled on semi-solid foods or beverages versus a traditional tablet supplement in pregnant women.

**Methods:**

Eighteen healthy pregnant women (24 – 32 weeks gestation) were randomized to receive the supplements in a crossover design. Following ingestion of each supplement, the changes (over baseline) in serum iron and folate over 8 hours were determined. The powdered supplement contained 30 mg of iron as micronized dispersible ferric pyrophosphate with an emulsifier coating and 600 μg folic acid; the tablet contained 27 mg iron from ferrous fumarate and 1000 μg folic acid.

**Results:**

Overall absorption of iron from the powdered supplement was significantly lower than the tablet (p = 0.003). There was no difference in the overall absorption of folic acid between supplements. Based on the differences in the area under the curve and doses, the relative bioavailability of iron from powdered supplement was lower than from the tablet (0.22).

**Conclusion:**

The unexpected lower bioavailability of iron from the powdered supplement is contrary to previously published reports. However, since pills and capsules are known to be poorly accepted by some women during pregnancy, it is reasonable to continue to explore alternative micronutrient delivery systems and forms of iron for this purpose.

**Trial Registration:**

ClinicalTrials.gov NCT00789490

## Background

Iron deficiency anemia (IDA) is the most common micronutrient deficiency in women of reproductive age affecting approximately 17% of women during pregnancy [1].

Supplements containing iron and folic acid are recommended during pregnancy by various health organizations to meet requirements and to reduce the risk for deficiency [2]. Folate deficiency has significantly decreased in North America primarily due to folic acid fortification of the food supply but the need for adequate folate intake prior to conception and during the early weeks of pregnancy remains a significant concern to reduce the risk for the development of neural tube defects (NTD) [3]. Approximately 57.7% of Canadian women will take a multivitamin preparation prior to conception and up to 89.7% will take multivitamins containing folic acid during pregnancy, primarily in the first three months of gestation. [4]. Up to half of the women taking multivitamin and iron supplements during pregnancy will experience some gastrointestinal side effects, particularly constipation and nausea [5-7], thus it is not surprising that adherence to supplementation during pregnancy is only about 50% [7]. This limited adherence to supplementation during pregnancy due to gastrointestinal side effects associated with supplemental iron can be further exacerbated by morning sickness and may also be related to tablet size [7-9].

A recent innovation in the delivery of minerals and vitamins has been a powdered form of iron and folic acid packaged in single-serve sachets that are sprinkled over any semi-solid foods just before consumption. The innovation has been coined 'point of use' or 'home'-fortification. 'Point of use' fortification was designed to improve adherence by reducing the side-effects of the iron through the use of microencapsulated ferrous fumarate as the iron source, as well as the buffering effect of the food to which the fortificant is added. The encapsulate is an edible vegetable- based lipid which dissolves in the low pH environment of the stomach. Microencapsulation masks the metallic taste of the iron and possibly protects the gastric epithelium from local irritation by the iron salt[10]. 'Point of use' fortification has been associated with a reduction in the incidence of anemia and is associated with improved adherence when provided to infants and children [11-14].

A disadvantage of using microencapsulated ferrous fumarate as the iron source in 'point of use' fortificants is that it has limited solubility (because of the lipid encapsulate) thus it is not readily suitable for use in beverages. To potentially improve adherence it would be of value to use a mixture of minerals and vitamins (including iron) that would readily disperse in liquids or semi-solids. A new compound SunActive Fe^® ^is a micronized dispersible form of ferric pyrophosphate (MDFP) with an emulsifier coating, which was designed to increase the bioavailability and solubility of ferric pyrophosphate. While ferric pyrophosphate has excellent organoleptic properties, the bioavailability is quite low compared to ferrous fumarate [15] The potential advantage of MDFP is that it dissolves readily in liquids or semi-liquid foods and is reported to have a relative bioavailability (RBV) of 82 – 92% depending on the food-based carrier [16].

There is no data on the absorption kinetics of MDFP and powdered folic acid when used in a multi-vitamin/mineral supplement in pregnant women. Thus, the objective of the current study was to evaluate the relative bioavailability and absorption kinetics for both iron and folic acid using the new 'point of use' fortificant containing 600 μg of folic acid and MDFP (SunActive Fe^®^) to supply 30 mg of iron, compared to a traditional pregnancy tablet supplement, which contains 1000 μg of folic acid and 27 mg of iron from ferrous fumarate.

## Methods

### Subjects

Twenty-six healthy women (aged 18 to 45 y) between 24 to 32 weeks gestation were recruited from staff and visitors at the Hospital for Sick Children, Toronto between December 2005 and June 2006. Eight subjects withdrew from the study due to pregnancy complications or time conflicts, thus 18 subjects were enrolled. Women were excluded if they had any of the following: significant complications of pregnancy; acute or chronic illness or diseases; any hematological disorders; any conditions that would interfere with the absorption, metabolism or excretion of iron or folic acid; on antibiotic therapy; known or suspected allergies to supplement ingredients; anemia (Hb<110 g/L) or elevated hemoglobin (= 144 g/L) or a blood transfusion 3 months prior to enrollment. Prior multivitamin/mineral supplement use did not preclude participation in the study. One subject with hemoglobin concentration of 106 g/L was inadvertently included into the study. However, the serum iron and folate results from this subject did not affect the study outcomes.

At baseline the mean (± SD) age of the subjects was 33 ± 4 years; gestational age 26.4 ± 2.8 weeks. None were smokers, one was a vegetarian and the majority (56%) had one child. All had single-birth pregnancies. At the time of recruitment, sixteen out of the eighteen women were taking daily supplements containing 27 mg iron and 1000 μg folic acid.

Clinical trial application approval was obtained from Health Canada and the study protocol was approved the Research Ethics Board of the Hospital for Sick Children and the University of Toronto. Consent from each subject was obtained on the first study visit and all data was coded to ensure confidentiality.

### Study Design and Methods

**Figure 1:**
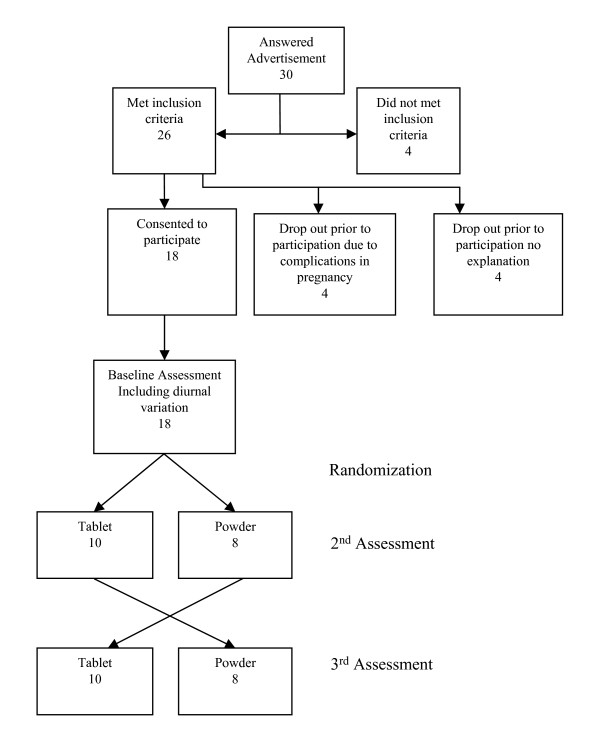
**Study Flow Diagram**.

This was a randomized, 3-period, 2-intervention crossover study. (Figure 1). Composition of the two supplements, are shown in Table 1. The powdered supplement was prepared using SunActive Fe^® ^and other micronutrients by Nealanders International Inc. (Mississauga Canada) and repackaged into 1 gram aliquots in white plastic Tamper Seal Vials (Pharmasystems). SunActive Fe ^® ^is a proprietary iron source produced by Taiyo Kagaku Ltd (Yokkaichi Japan). The control supplement, Materna^® ^is manufactured by Wyeth Consumer Healthcare Inc (Mississauga Canada). The form of iron in the traditional tablet supplement is ferrous fumarate.

**Table 1:** Supplement composition

	**Materna**^**®**^ **per 1 tablet**	**Powdered Supplement** **per 1 g dose**
Vitamin A, μg RE	1500	800
Vitamin C, mg	100	70
Vitamin D, μg	6.4	5
Vitamin E, mg a-TE	30	15
Vitamin B1, Thiamin mg	3	1.4
Vitamin B2, Riboflavin mg	3.4	1.4
Vitamin B6, Niacin mg	10	1.9
Vitamin B12, μg	12	2.6
Folic Acid, μg	1000	600
Niacin, mg NE	20	18
Iron, mg ^1^	27	30
Zinc mg	25	5.5
Copper, mg	2	1
Iodine, μg	150	220
Beta-Carotene, unit	1500	0
Biotin, μg	30	30
Calcium (Calcium Carbonate), mg	250	0
Chromium (Chromic Cloride), μg	25	0
D-Pantothenic Acid, mg	10	6
Magnesium (Magnesium oxide), mg	50	0
Manganese (Manganese Sulphate), mg	5	0
Molybdenum (Sodium Molybdate), μg	25	0
Selenium (Sodium Selenate), μg	25	0

Table shows the composition of the tablet and powdered supplements. The iron sources for the supplements are ferrous fumarate in the tablet and micronized dispersible ferric pyrophosphate in the powdered supplement.

Subjects attended a baseline and two subsequent assessment visits, each one week apart. Prior to each assessment, subjects fasted overnight (for 10 hours) and refrained from taking their usual supplements. Subjects were allowed to take their usual supplements between each assessment visit. During each assessment an indwelling intravenous catheter was inserted and six venous blood samples (5 ml each) were drawn at 0, 1, 2, 3, 4 and 8 hours after supplement ingestion. Blood samples were collected without and with the anticoagulant EDTA for latter determination of iron and folate biochemical indices, respectively. The tubes for folate analysis were covered with foil to block light. Serum and plasma were separated from whole blood by centrifugation (1500 RPM for 15 min at 4°C). One percent sodium ascorbate was added to stabilize the folate. Samples were aliquoted and frozen at -80°C until analysis.

All subjects participated in an initial assessment during which neither the tablet nor the powdered supplement were provided. During the initial assessment, hematological status was determined as well as serum iron diurnal variation by determining serum iron concentrations over the 8-hour assessment period (as described above). Since there is no discernable diurnal variation for folate, plasma folate was not measured during the initial assessment [17]. For the second and third assessment periods, the subjects were randomly assigned (by drawing coloured poker chips from a bag) to either the tablet supplement or the powdered supplement which the subjects were allowed to sprinkle either onto a semi-solid food such as applesauce or a liquid such as juice prior to consumption. If the tablet supplement was ingested during the second assessment period, then the powdered supplement would be provided during the third assessment period and vice versa. Supplements were taken with a standardized morning snack, consisting of oatmeal cookies, apple sauce and juice, after the first blood draw. The morning snack contained 1.5 mg of iron, 28.2 μg folic acid, and 88.0 mg of ascorbic acid and no calcium or caffeine. Nutrient values of the snack were determined either from product labels or the Canadian Nutrient File [18]. Ascorbic acid was incorporated into the standardized morning meal to enhance iron bioavailability. A standardized low iron, low folic acid lunch was consumed at the 4^th ^hour of each, assessment period. Subjects were encouraged to consume all their food and the complete consumption of the powdered supplement. The same food was consumed for all three assessment periods. Changes in serum concentrations of iron and folate were measured for each subject during each of the two 8-hour assessment periods.

### Assessment of Baseline Iron and Folate Status

Hemoglobin (Hb) concentration was measured prior to insertion of the intravenous catheter to determine study eligibility. Hb concentration was determined directly from capillary blood via finger prick and measured using a portable HEMOCUE B-Hb photometer (Hemocue, Angelholm, Sweden) by the trained study coordinator using standardized techniques. Initial blood samples on the first assessment day were used to determine serum ferritin concentration and serum transferrin receptor (sTfR) for overall hematological status. Both ferritin and serum transferrin receptor were measured using standard laboratory methods [19-21].

### Serum Iron 'Area Under the Curve' (AUC)

Serum iron was measured using a colormetric assay (Vitros Chemistry) with a quality control precision of 17.1 ± 0.35 μmol/L and CV of 3.1% [22,23]. Hemolyzed samples were discarded. The changes in serum iron concentrations over time were used to calculate the area under the plasma curve (AUC_0–8h_) using the linear trapezoid rule [24]. Total area under the curve for basal diurnal variation was measured from the concentrations of the serum iron during the baseline assessment. Thus to account for the change in serum iron due to diurnal variation, the baseline iron measurements at each time point were subtracted from those measured during assessment periods 2 and 3. The adjusted area under the curve (for assessment periods 2 ands 3) was calculated as follows:

^adjusted^AUC[*i*, *j*] = AUC[*i*, *j*] - mean baseline AUC [Adapted from Hoppe et al [25]]

Where *i *indicates participant and *j *indicates intervention (powdered supplement or tablet) and the mean baseline AUC for diurnal variation is the mean AUC value across all subjects from the baseline visit as per Hoppe et al [25]. The individual baseline (diurnal variation) results were also used to calculate the AUC; however this did not influence the statistical results. Relative bioavailability (defined as the measure of bioavailability of the iron in the powdered supplement against bioavailability of the iron in the tablet {AUCa * dose b}/{AUCb * dose a} was calculated.

### Plasma Folate 'Area Under the Curve' (AUC)

As previously mentioned, since there is no discernable diurnal variation for folate, plasma folate was not measured during the baseline assessment on the initial assessment day [17]. Total folate concentration was assessed using the microbiological assay as described by Molloy and Scott [26] using *Lactobacillus rhamnosus *(ATCC 7649; American Type Tissue Culture Collection, Manassas, VA) as the test organism. All samples from the same subjects were analyzed at the same time to reduce intra-subject variability. A whole blood folate standard prepared by the National Institutes for Biological Standards and Control (NIBSC code 95/528, certified content of 29.51 nmol/L (13 ng/ml), NIBSC Hertfordshire EN6 3QG United Kingdom) was used to assess the accuracy and precision of the folate bioassay (a plasma folate certified standard does not exist). The overall inter-assay coefficient of variation for the whole blood folate standard was 9.1% with a mean value of 32.2 ± 2.9 nmol/L (14.2 ± 1.3 ng/ml). Incremental AUC was measured during each assessment period for each subject. Changes in plasma folate concentration at each time period were calculated by subtracting the baseline (the 08:00 AM value) from the subsequent values.

### Statistics

A sample size calculation estimated that 16 women were required to detect a statistically significant difference of 36 in AUC between the two groups (80% power and SD of 46). The estimated mean and SD for AUC were based on previous literature [25]. A similar calculation was done for folate which projected a sample size of 18 subjects to detect a 20% difference in mean folate AUC with an α = 0.05 and 80% power [27]. To account for uncertainty imposed by pregnancy and blood sampling, a 20% allowance was added to the estimated sample size, thus 19 women were originally planned for recruitment. All data collection sheets and lab reports were manually checked for completeness and accuracy of data. Results from one subject was excluded since iron concentration values from the tablet supplement were greater than three standard deviations above the overall distribution mean (p > 0.05). A parametric analysis was performed with a repeated measures analysis of variance model and Satterthwaite formula to estimate degrees of freedom. The fixed effects in the model were: age, gestational age, ferritin and parity. Subject was included as the repeated effect. The pair-wise differences of least squares means of the interventions were tested using Tukey-Kramer p value adjustments. All statistical tests were 2-tailed and a p-value of ≤ 0.05 was considered statistically significant. Statistical software SAS (version 9.1, SAS Institute Inc. Cary, North Carolina) was used for the analysis.

## Results

Results for hematologic and folate status are shown in Table 2. Seventy-Two percent of plasma folate values were above the normal range at baseline, while 50% of ferritin values were below reference normal values for pregnant women. Normality of variables was tested and found to be not significant for Kolmogorov-Smirnov test with the exception of ferritin. Ferritin was log transformed prior to analysis.

**Table 2:** Baseline measures of iron and folate status under fasting conditions

	**Mean ± SD** **N = 18**	**Range**	**% outside normal range**	**Direction**
Hemoglobin g/dL	12.6 ± 0.8	10.6–13.7	0*	
Serum Ferritin μg/L	14.1 ± 8.3	4.5–31.4	50**	below
sTfR mg/L	3.2 ± 0.9	1.9–5.5	5.5***	above
Serum Iron μmol/L	14.0 ± 5.7	6.7–25.5	0****	
Plasma folate nmol/L	57.2 ± 19.2	17.6–102.2	72*****	above

*n *= 18. sTfR, serum transferrin receptor. *Hb cutoff during 2^nd ^trimester is defined as 105 g/L and 110 g/L during third trimester[44] **Low ferritin values defined as <12 μg/L [43]. ***High sTfR values defined as 4.9 mg/L as per kit [21]. Serum iron for non-pregnant women is defined as 6.7–30.4 μmol/L[23] **** High serum folate values defined as >45.3 nmol/L [41].

### AUC Measures for Iron

**Figure 2:**
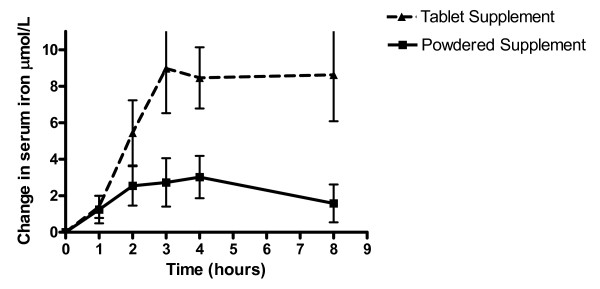
**Mean incremental changes in serum iron concentrations between tablet and powdered supplements**. Mean (± SEM) incremental changes in serum iron concentration in pregnant subjects over 8 hours after administration of either 27 mg of iron from ferrous fumarate in a traditional tablet supplement or 30 mg of iron from micronized dispersible ferric pyrophosphate in powdered supplement sprinkled over a standard meal. *n *= 17. The curve was adjusted for basal (diurnal) variation and the iron content of the standardized meal. There was a significant difference (p = 0.0003) between the relative bioavailability (as measured using AUC) of the iron in tablet supplement (41.8 ± 45.9 μmol·h/L) when compared to the iron in the powdered supplement (10.0 ± 43.3 μmol·h/L). The data were analyzed with the use of mixed-model repeated-measures with age, gestational age, ferritin concentration and parity as fixed effects and subject as the repeated effect. The pair-wise differences of least-square means of the treatments were tested with the use of Tukey-Kramer p value adjustments.

For each subject, baseline serum iron values did not significantly differ within subjects or between groups for all three assessment periods. Values for basal diurnal variation AUC, adjusted AUC, peak concentration and time to peak by treatment are shown in Table 3. Time to peak and peak concentration were computed via the software program (GraphPad Prism, version 4.0 2003, GraphPad Software Inc, La Jolla, CA). Net changes in mean serum iron concentrations are shown in Figure 2. There was a statistically significant difference in the change in serum iron between supplements at 3- (p = 0.026), 4- (p = 0.0083) and 8-hours (p = 0.0071) post ingestion. The AUC -iron was significantly smaller for the powdered supplement than for the tablet (p = 0.0003). Relative bioavailability for micronized dispersible FePP in powdered supplement was 22% compared to the ferrous fumarate in tablet supplement.

**Table 3:** Area under the curve, basal diurnal variation (DV), serum iron and plasma folate by treatment

	Basal Diurnal Variation (DV)	Powdered Supplement	Tablet Supplement
**Iron**			
AUC (μmol·h/L)	104.5 ± 34.1		
Adjusted AUC(after DV) (μmol·h/L)		10.0 ± 43.3*	41.8 ± 45.9*
Peak value (μmol/L)	14.6	3.0	8.9
Time to peak value (hrs)		3	4
			
**Folate**			
Incremental AUC (nmol·h/L)	NA	271.8 ± 110.9	248.7 ± 140.2
Peak value (nmol/L)	NA	54.4	53.1
Time to peak value (hrs)	NA	4	3

AUC, area under the curve. All values are mean ± SD; *n(iron) *= 17; *n(folate) *= 18 (sample size differed according to subject exclusion for iron variables). *p = 0.0003.

### AUC Measure for Folate

**Figure 3:**
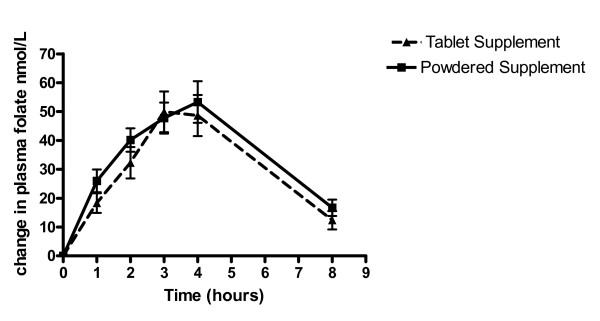
**Mean incremental changes in plasma folate concentrations between tablet and powdered supplements**. Mean (± SEM) changes in plasma folate concentrations in pregnant subjects over 8 hours after administration of either 1000 μg folic acid in the traditional tablet supplement or 600 μg folic acid in the powdered supplement sprinkled over a standard meal. *n *= 18. There was no significant difference in the area under the folate absorption curve in the tablet supplement (248.7 ± 140.2 nmol·h/L) when compared to the folic acid in the powdered supplement (271.8 ± 110.9 nmol·h/L). The data were analyzed with the use of mixed-model repeated measures with age, gestational age, ferritin concentration and parity as fixed effects and subject as the repeated effect. The pair-wise differences of least-square means of the treatments were tested with the use of Tukey-Kramer P value adjustments.

Baseline (8 am sample) mean plasma folate concentrations did not differ within or between subjects. Values for folate by intervention are shown in Table 3. AUC for folate was not related to any of the other study variables (Ferritin concentration, age, gestational age). Net changes in mean plasma folate concentrations are shown in Figure 3. No significant differences were observed (p = 0.61) in the area under the folate curve in the traditional tablet supplement and in the new powdered supplement. There was no difference in the peak absorption of either supplement.

## Discussion

Given the documented importance of vitamin and mineral supplementation during pregnancy and the relatively poor adherence to currently available supplements, our goal was to develop a supplement that was efficacious in terms of iron and folic acid bioavailability, yet could potentially result in improved adherence. Thus, we investigated the absorption kinetics of a micronized dispersible form of ferric pyrophosphate (MDFP) with an emulsifier coating and folic acid in a powdered multivitamin supplement in healthy pregnant women versus ferrous fumarate (non-encapsulated) in a traditional tablet supplement. Both iron and folic acid were absorbed from the powdered supplement, however based on the differences in the area under the curve (AUC) and doses, the relative bioavailability of iron from powdered supplement was lower than from the tablet supplement (0.22), while folic acid was greater (1.8).

In children, powdered supplements which included encapsulated ferrous fumarate as the iron source were shown to be equally as effective as ferrous sulfate drops at reducing the incidence of iron deficiency anemia but with improved adherence [10-14]. Using stable isotopes, it was directly demonstrated that 8.3% of the encapsulated ferrous fumarate was absorbed in iron-deficient anemic children compared to 4.3% in iron deficient and iron sufficient children [28]. The MDFP in the powdered supplement in the present study had a distinctly different absorption curve from the ferrous fumarate in the tablet supplement. It is possible that the emulsifier coating on the MDFP delayed or reduced absorption of the iron by keeping the iron suspended within the gastric lumen for a longer period of time compared to the iron in the tablet thereby increasing its interaction with dietary inhibitors or allowing the MDFP to exit the stomach and duodenum without being dissolved [29]. Other factors may also have influenced the bioavailability of the iron in the powdered supplement. Navarro et al [27,30] showed distinctly different curves in the absorption kinetics of iron in the comparison of a whole versus crushed tablet with the crushed tablet having a lower absorption than the whole tablet. It is possible that the smaller particle size in the crushed form may enhance micronutrient interactions resulting in reduced absorption of the iron, whereas the tablet form may offer some protection from dietary inhibitors. Alternatively, the combination of powder form and emulsifier coating could delay both the reduction of a portion of the ferric form of iron to the ferrous form by dietary acids as well as the transport of the ferric iron into the enterocyte by the Integrin-Mobilferrin pathway [29].

Ferric pyrophosphate is significantly less bioavailable than more water-soluble iron compounds, however decreasing the particle size of the ferric pyrophosphate has been shown to improve its bioavailability [16]. It has been suggested that the relative bioavailability of MDFP may be more influenced by the food matrix and other dietary inhibitors than more soluble forms of iron [16,29]. Hallberg et al [31] found that the relative bioavailability of elemental iron powders could be markedly affected depending on the food matrix within which they were studied possibly due to different rates of dissolution with different meals. In the current study, it is likely that the food matrix had a more significant effect on the gastric dissolution of the SunActive Fe in the powdered supplement than it did on the ferrous fumarate in the tablet. Ascorbic acid acts as both a reducer and a chelator in the intestinal lumen to promote iron absorption. Previous studies have found that addition of ascorbic acid had less of a promoting effect on the absorption of ferric pyrophosphate than it did on ferrous sulfate, decreasing the relative bioavailability of ferric pyrophosphate [29,32]. In the present study, both the tablet and the powdered supplement contained ascorbic acid (100 vs 70 mg respectively) and an additional 88 mg was consumed at breakfast when the supplements were taken. It is possible that the addition of ascorbic acid from both the supplement and the food matrix promoted the absorption of the ferrous fumarate in the tablet supplement more than it promoted the absorption of the MDFP in the powder supplement. Calcium and zinc (at high doses) have been noted to depress iron absorption [33,34]. Despite the fact that the powdered supplement contained no calcium and a low dose of zinc, iron absorption was not enhanced. We assume that the impact of the food matrix had the strongest impact on iron absorption.

Given the lower relative bioavailability of the iron in powder supplement, MDFP may not be the best iron source for the powder supplement. MDFP was chosen because it causes less organoleptic changes to foods than ferrous fumarate and it is dispersible in liquids due to its emulsifier coating. Initial studies using erythrocyte incorporation methodology demonstrated that it was as well absorbed as ferrous sulfate [16]. In addition, we recently reported significant and similar increases in hemoglobin and ferritin concentrations among children randomized to either powdered ferrous fumarate or MDFP at varying doses compared to ferrous glycine sulfate drops after an eight 8 week intervention [35]. This study however, did not attempt to measure the impact on iron status of the different iron compounds.

The relative bioavailability of the folic acid was greater in the powdered form than the tablets despite the higher dosage in the tablet (1000 vs. 600 μg). This observation supports earlier evidence that the absorption of folic acid may be blunted at high oral intake. The relative response of folic acid seems to be more effective at lower doses and absorption curves have been shown to plateau at higher doses [36]. Doses of folic acid above 200 μg have resulted in the appearance of unmetabolized folic acid in serum [37]. Houghton et al [38] found no evidence of further RBC folate increase above an oral intake of 475 μg while Caudill et al [39] found no difference in RBC folate between 450 and 850 μg of folate per day. Wright et al [40] found that folic acid does not appear to be reduced in the intestine as previously thought but rather appears to enter the hepatic portal vein unaltered and is sequestered and metabolized in the liver. Since the liver has a limited capacity to convert folic acid to dihydrofolate, a form utilized by the body, significant amounts of folic acid can enter circulation [40]. Wright also showed that the plasma response to a folate containing meal included endogenous folate as well as the folate in the meal. [40]. However, we demonstrated no difference in relative bioavailability despite the difference in doses. In the current study, mean plasma folate from the entire cohort was above the reference normal range [41]. These elevated levels suggest that the folic acid intake from both supplements exceeds the metabolic capacity of the liver cells and would likely contribute to the presence of unmetabolized folic acid in maternal serum (although this was not measured in the current study). These high intakes could be exposing developing fetuses to undesirable levels of unmetabolized folic acid for which the long-term consequences are unknown [42]. The results of the current study support the current WHO recommendation of including no more than 400 μg of folic acid in prenatal supplements, particularly in countries that mandate folate fortification [43].

This study only measured relative bioavailability, therefore it was not possible to determine how much of the micronized dispersible ferric pyrophosphate was absorbed and whether it would be sufficient to maintain iron status during pregnancy. Further investigation is needed to determine whether this form of iron is effective at maintaining iron status during pregnancy. Although not anticipated, about half of the subjects were iron depleted at baseline even though hemoglobin was within normal range for pregnancy[44]. Thus extrapolation of the results to an iron-replete population may not be appropriate. It is suggested in the literature that low iron stores should have actually increased the absorption of the iron from the supplements rather than decreasing it[45]. However, we showed no correlation between the AUC for serum iron and ferritin likely due to the small sample size of the study. Similarly, this study was not designed to examine the adherence of a powdered mineral supplement versus traditional tablets, nor the efficacy of one versus the other. Both the iron and folic acid were absorbed from the powdered supplement, although the iron in the powdered supplement was significantly less bioavailable than the iron in the tablet supplement and folate was more bioavailable from the lower-dose powder supplement. While our ability to extrapolate the relative bioavailability of the iron was impeded by the curve not approaching baseline, it is evident that the iron in the powdered supplement did not perform as expected. Further clinical studies would need to be conducted to determine both the efficacy and effectiveness of the new powdered supplement in pregnant women.

## Conclusion

The lower bioavailability of the iron in the powdered supplement was unexpected and contrary to previously published reports. Since pills and capsules are known to be poorly accepted by some women during pregnancy, especially those with significant morning sickness, we believe it is reasonable to continue to look for alternative delivery systems and forms of iron for this purpose.

## Competing interests

Dr. Stanley Zlotkin has beneficial interests in certain intellectual property rights to his invention known as "Sprinkles". These interests include (i) patent rights for the United States and Canada which are held by Ped-Med Limited, a Canadian corporation, of which Dr. Zlotkin is the sole shareholder and (ii) trade-marks rights in various jurisdictions to the name "Sprinkles" which are held either by Ped-Med Limited or by the Sprinkles Global Health Initiative Inc. a Canadian not-for-profit corporation of which Dr. Zlotkin is a member. The remaining authors declare that they have no competing interests.

## Authors' contributions

BHC, AC and SZ were responsible for the study concept and design. BHC was responsible for conducting the study and the acquisition and analysis of all data. DLO was responsible for the folate component of the study and interpretation of the data pertaining to folate. BHC was responsible for conducting the microbiological assay under supervision of DLO. Drafting of the manuscript was performed by BHC and SZ. All authors read and approved the final manuscript.

## Pre-publication history

The pre-publication history for this paper can be accessed here:


